# More Stable Productivity of Semi Natural Grasslands than Sown Pastures in a Seasonally Dry Climate

**DOI:** 10.1371/journal.pone.0035555

**Published:** 2012-05-09

**Authors:** Sonia Ospina, Graciela M. Rusch, Danilo Pezo, Fernando Casanoves, Fergus L. Sinclair

**Affiliations:** 1 Area of Sustainable Livestock, Center for Research in Sustainable Agricultural Production Systems, Cali, Colombia; 2 Department of Terrestrial Ecology, Norwegian Institute for Nature Research, Trondheim, Norway; 3 Livestock and Environment Program, Tropical Agricultural Research and Higher Education Center, Turrialba, Costa Rica; 4 Biometric Unit, Tropical Agricultural Research and Higher Education Center, Turrialba, Costa Rica; 5 College of Natural Sciences, Bangor University, Wales, United Kingdom and Production Ecology, World Agroforestry Centre, Nairobi, Kenya; MESC; University of South Alabama, United States of America

## Abstract

In the Neotropics the predominant pathway to intensify productivity is generally thought to be to convert grasslands to sown pastures, mostly in monoculture. This article examines how above-ground net primary productivity (ANPP) in semi-natural grasslands and sown pastures in Central America respond to rainfall by: (i) assessing the relationships between ANPP and accumulated rainfall and indices of rainfall distribution, (ii) evaluating the variability of ANPP between and within seasons, and (iii) estimating the temporal stability of ANPP. We conducted sequential biomass harvests during 12 periods of 22 days and related those to rainfall. There were significant relationships between ANPP and cumulative rainfall in 22-day periods for both vegetation types and a model including a linear and quadratic term explained 74% of the variation in the data. There was also a significant correlation between ANPP and the number of rainfall events for both vegetation types. Sown pastures had higher ANPP increments per unit rainfall and higher ANPP at the peak of the rainy season than semi-natural grasslands. In contrast, semi-natural grasslands showed higher ANPP early in the dry season. The temporal stability of ANPP was higher in semi-natural grasslands than in the sown pastures in the dry season and over a whole annual cycle. Our results reveal that, contrary to conventional thinking amongst pasture scientists, there appears to be no increase in ANPP arising from replacing semi-natural grasslands with sown pastures under prevailing pasture management practices in seasonally dry climates, while the temporal distribution of ANPP is more even in semi-natural grasslands. Neither sown pastures nor semi-natural grasslands are productive towards the end of the dry season, indicating the potential importance of the widespread practice of retaining tree cover in pastures.

## Introduction

The most widespread type of pasture in the humid and sub-humid Neotropics is semi-natural grassland derived from the clearing forests [1], [2], [3]. The grasslands are mainly unsown herbaceous communities that we refer to as semi-natural grasslands, because their plant communities are composed mainly of native species, with a predominance of various prostrate grasses of the genus *Paspalum* and *Axonopus*, and are maintained by grazing and the removal of much of the encroaching woody vegetation. Their large extent implies that they have a significant role in the global carbon cycle but their contribution is not well understood because of the paucity of high quality data on primary productivity and limited information about their ecological characteristics[4], [5]. In most tropical America these pastureland types occur in areas where the natural vegetation has been classified as seasonally dry forest, characterized by a strong seasonal growth pattern determined by the distribution of rainfall [6].

Primary productivity and total rainfall are known to correspond closely in the sub-humid and arid regions [7], [8] and long-term data show a significant linear relationship between those variables for many sites around the world [7], [9], [10]. An understanding of the response of above-ground net primary productivity (ANPP) to rainfall becomes especially important in view of global warming. For Central America, scenarios for the dry and wet seasons predict a relative decrease in rainfall of 10 and 20%, respectively, for the period 2090–2099, relative to 1980–1999 [11].

In seasonally dry climates, primary productivity is affected not only by the total annual or seasonal rainfall, but also by rainfall distribution [12], [13], [14]. For semi-arid systems it has been suggested that the majority of the primary productivity occurs in the form of short-duration pulses following rainfall events [15] and in a mesic tall-grass prairie, Knapp and co-workers [16] found that when the total rainfall for the season was kept constant, extending the dry interval between rainfall events reduced ANPP by about 10%; but, such correlation between primary productivity and distribution of rainfall has not been well established for tropical sub-humid regions. Specifically in Central America, there is little published information about inter-seasonal variations of rainfall components (total amount, number of rain events, rain event size, length of dry intervals), but some evidence from climatically similar regions shows that seasonal rainfall distribution is more closely correlated to primary productivity than the overall amount of seasonal rainfall [17]. In five tropical regions including the Brazilian Northeast, inter-annual variations in the number of rainy days within a season were more highly correlated with ANPP than fluctuations in the total seasonal rainfall [18], but it remains unclear whether different rainfall components have varying effects depending on the kind of vegetation cover.

At local level, factors other than climate, including the composition of vegetation, become important determinants of productivity [19], but whether different rainfall components have varying effects depending on the kind of vegetation cover still remains unknown. This knowledge is important, particularly because human interventions often change the vegetation cover. Both community composition and vegetation cover may alter the relationship between productivity and annual rainfall [10], [20] therefore site-specific models are required for effective prediction of production in response to climate [10].

In Central America, as in other areas with sub-humid and seasonally dry climates, savannas and grasslands are often replaced by sown pastures with the aim of increasing the amount and quality of feed offered to cattle [21]. In the area of the research reported here, this conversion involves the replacement of the vegetation cover but frequently with no changes in resource supply (water and nutrients). Ecological theory predicts that changes in the composition and diversity of communities may have significant impacts on ecosystem function in terms of process rates, biomass and element pool sizes and variation, measured by the extent of their fluctuation [22]. The effects of community composition are often linked to particular attributes of the dominant species and to how these relate to resource acquisition rates [23], [24] and their response to disturbance [25].

Higher productivity can be expected with higher diversity because diverse communities are likely to include combinations of species that are functionally complementary [22]. Co-existing species in savannas and grasslands in the Neotropics reach peak biomass at different times of the season [1]. These patterns could be explained by specific differences in traits that determine plant responses to between- and within-seasonal variations in rainfall. In contrast, monocultures of cultivated varieties could be expected to be more productive over short periods of time and at small spatial scales, but probably not during a complete year or at larger spatial scales [26], [27], [28] because in monocultures, productivity is essentially channelled through a single life form and the process of domestication and selection acts on a subset of attributes, narrowing the genetic pool [29]. Often in seasonally dry environments inputs such as fertilizer and/or irrigation are required for monocultures to be productive.

Whereas annual ANPP is a measure of the annual ecosystem function [30], the variability of ANPP through the season provides an insight into how an ecosystem responds to fluctuations in rainfall within a year [14]. Variability in productivity has been strongly associated with grassland stability [31], and through the concept of community temporal -stability, linked to species diversity [32].

The research reported here focuses on the temporal variability of ANPP within a range of semi-natural grasslands (hereinafter, grasslands) and sown pastures. An insight into the amount and pattern of primary productivity of the spontaneous and introduced vegetation common to the sub-humid regions of Central America, and their relationships with rainfall seasonality, is essential for understanding the consequences of transforming grasslands into sown pastures; and the consequences of such transformations in view of expected shifts in rainfall patterns anticipated as a result of global warming.

We hypothesized that sown pastures would have higher ANPP in the peak of the rainy season, but grasslands would start growth earlier in the season and continue longer into the dry season, when rainfall events are more erratic. We specifically aimed to find out: (i) whether ANPP in sown pastures and grasslands was related to rainfall and its distribution over short time periods; (ii) which rainfall parameters best explained the variation in ANPP; (iii) whether the ANPP of sown pastures was higher than for grasslands, when rainfall parameters in the dry and the rainy seasons were controlled for; (iv) whether ANPP was more stable through time for grasslands than sown pastures; and (v) what was the annual ANPP for grasslands and sown pastures under the prevalent conditions in the study.

## Results

### Summary of Current Rainfall Trends

The annual rainfall recorded in 2007 and 2008 was 1638 and 1858 mm, respectively. In 2007, the rainy season, (6^th^ June–30^th^ November) accounted for 83% of the annual rainfall. In 2008, the rainy season lasted from May to October comprising 1506 mm, of which 71% was concentrated in the first trimester (28^th^ May–28^th^ August). The start of the rainy season in 2008 was at the end of May. The cumulative rainfall for the month was 95 mm, but 75% of it occurred in the last four days of the month.

### Above-ground Net Primary Productivity, Rainfall and its Distribution

**Figure 1:**
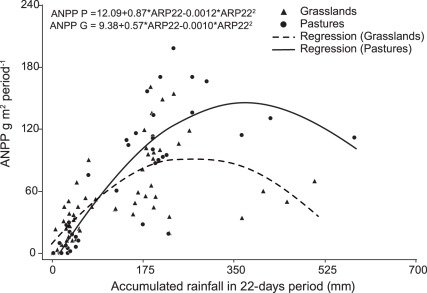
Relationships between above-ground net primary productivity and rainfall. The relationship between above-ground net primary productivity (ANPP) and cumulative rainfall in 22 day periods (ARP22) for sown pastures and grasslands. Sown pastures: n = 44, adjusted r^2^ = 0.74; grasslands: n = 60, adjusted r^2^ = 0.47. The relationships show significant linear and quadratic trends for pastures, in both cases, and grasslands. There were no differences between treatments in the intercepts and the quadratic coefficients but there were differences in the linear coefficient.

Overall, the frequency of rain events was lower in the 2007 than in the 2008 rainy season (Table 1). No significant differences in the amount of rainfall (*p* = 0.56) or in the number of rainfall events (*p* = 0.19) were detected between grasslands and *Brachiaria brizantha* pastures (hereon, ‘sown pastures’) sites. The relationships between ANPP and cumulative rainfall in each sampling period of 22 days (ARP22) show significant linear and quadratic trends for pastures (*p*<0.0001, in both cases) and grasslands (*p*<0.0001, in both cases). There were no differences between treatments in the intercepts (*p* = 0.0770), and the quadratic coefficients (*p* = 0.5564), but there were differences in the linear coefficients (*p* = 0.0228) (Figure 1). Under 300 mm of ARP22, the increase in ANPP with rainfall was larger in sown pastures than in grasslands but there was no difference between inflection points, indicating a similar rate of decline of ANPP in both treatments with higher ARP22. Then, we tested the contribution of the different rainfall components (cumulative rainfall and rainfall distribution metrics) in explaining ANPP variance in the linear portion of the ANPP vs. ARP22 regression curve. The relationship between ARP22 and the number of rainfall events (NRE), and ANPP was significant (adjusted R^2^ = 0.67; *p*<0.0001). ANPP in sown pastures increased with cumulative rainfall at higher rates, than grasslands (*p* = 0.0008). Annual ANPP was also significantly related to NRE (*p* = 0.0045) but there were no differences in this relationship between vegetation types (*p* = 0.1914). The other rainfall distribution variables tested (the size of the rainfall event and the interval between rainfall events) were not significantly related to ANPP.

**Table 1:** Mean cumulative rainfall and its seasonal distribution statistics.

Seasons	Type of ground vegetation cover	ARP22 (mm)	Mean number of rainfall events	Mean size of rainfall events (mm)	Mean interval between rainfall events (days)
Rainy Season 2007(July–November)	Grasslands (n = 4)	175	10	18	2
	S.D.	48	2	5	1
	Pastures (n = 3)	196	11	19	2
	S.D.	49	2	7	1
Dry Season 2007/2008 (December 2007–May 2008)	Grasslands (n = 4)	32	4	9	7
	S.D.	16	2	9	5
	Pastures (n = 3)	28	4	8	8
	S.D.	15	2	6	5
Rainy Season 2008(May–August)	Grasslands (n = 4)	261	11	22	2
	S.D.	130	3	7	1
	Pastures (n = 3)	294	12	24	3
	S.D.	155	3	9	2

Average rainfall in all of the 22 day periods and its distribution metrics in grasslands and sown pastures sites, during the rainy season 2007, dry season 2007/2008 and the initial trimester of the rainy season 2008, the period of measurement of above-ground net primary productivity and three rainfall distribution metrics: mean number of rainfall events, mean size of rainfall events (mm) and mean interval between rainfall events (days).

n = the number of sampled rain gauges contributing to the mean.

S.D. = standard deviation.

### Seasonality of Above-ground Net Primary Productivity

**Figure 2:**
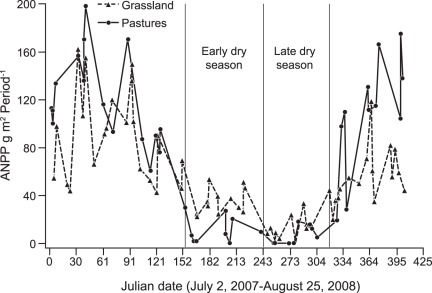
Above-ground net primary productivity through time. Variation of above-ground net primary productivity (ANPP in g m^2^ 22 day period^−1^) through time for grasslands and sown pastures. Sown pastures: n = 44; grasslands: n = 60. Black vertical lines separate seasons. The dry season was divided into early and late phases denoted by the grey vertical line.

**Figure 3:**
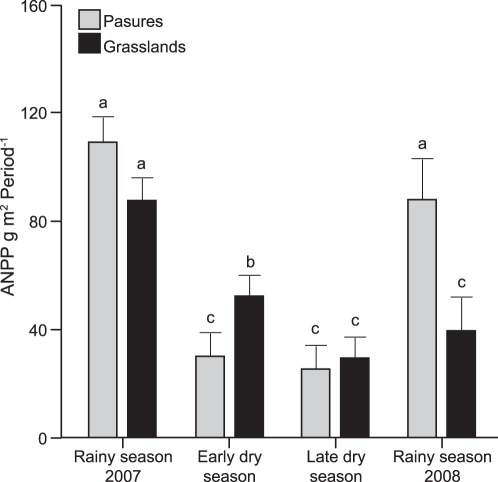
Above-ground net primary productivity for vegetation types and seasons. Mean above-ground net primary productivity (ANPP) for the different vegetation types in different seasons ± 1 SE, based on LSD test. Significant differences (*p*<0.05) between treatments are indicated by different letters.

**Figure 4:**
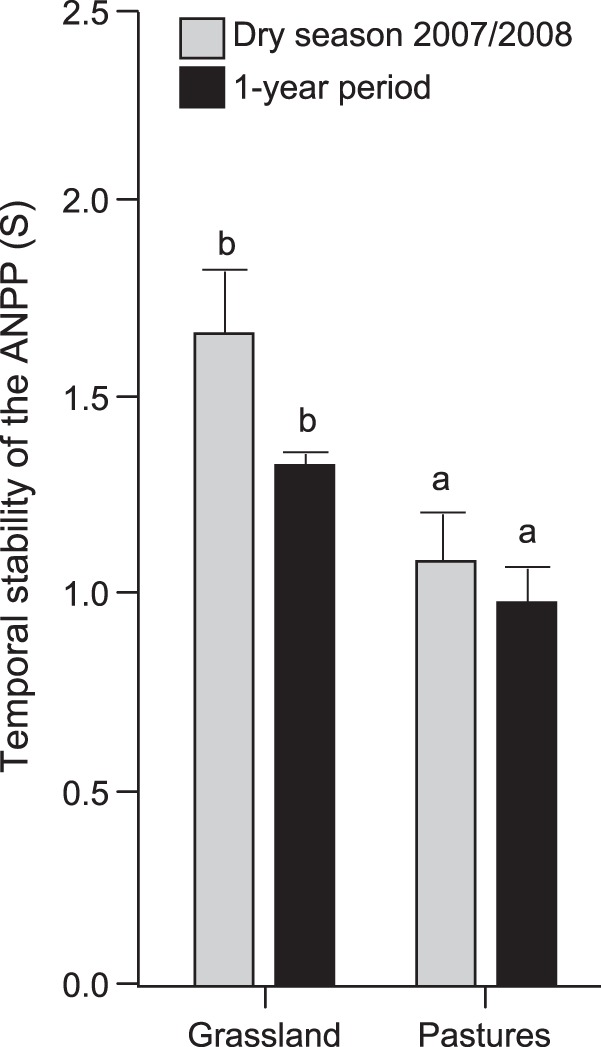
Temporal stability of Above-ground net primary productivity. Yearly (July 2007–June 2008) and dry season (December 2007–May 2008) temporal stability of ANPP, estimated in five plots with grasslands and at four plots with sown pastures in a sub-humid region of Central Nicaragua. Bars denote ±1 SE, based on LSD test. Different letters in the same time period indicate significant differences (*p*<0.05) between grasslands and sown pastures.

After adjusting ANPP by ARP22 and ARP22 squared there was a significant vegetation type x season interaction (F = 10.47, *p* = <0.0001), as well as significant main effects (vegetation type: F = 4.04, *p* = 0.0338, and season: F = 113.10, *p*<0.0001). The smallest variation corresponded to the late dry season 2007 and the highest to the rainy season 2008. Sown pastures had higher ANPP in the rainy season 2008, while grasslands had higher ANPP in the early dry season 2007 (Figures 2 and 3). The temporal stability of ANPP was higher in grasslands than in sown pastures for the dry season (F _6.3131_; n = 9; *p* = 0.0402) and the annual cycle (F _11.0474_; n = 9; *p* = 0.0127) (Figure 4). ANPP was very low for both sown pastures and semi-natural grasslands in the latter part of the dry season. The annual ANPP was 898 and 955 g m^−2^ yr^−1^ for grasslands and pastures, respectively.

## Discussion

### Relationships between ANPP, Rainfall and its Distribution

ANPP was positively associated with cumulative rainfall over 22 day-periods (ARP22). A positive relationship between ANPP and rainfall is consistent with empirical evidence from grasslands and savannas worldwide showing a correlation between ANPP and variability between- [6], [7], [9] and within- years [14], [33] in the amount of rainfall. In arid climates, ANPP and rainfall are linearly correlated, indicating that under conditions water availability is the key limiting factor for ANPP. In our study, a quadratic model resulted in a better fit than a linear. ANPP was less when the cumulative rainfall in a 22-days period (ARP22) was higher than ca. 300 mm. Apparently, the timing and intensity of the rainfall events were important in determining when ANPP increased with rainfall and at which point ANPP was low despite high cumulative rainfall.

The intensity of rainfall events is an important determinant of infiltration [34] and in turn soil water contents and productivity. Low water retention and lower productivity per unit rainfall have been observed in grasslands of various conditions and composition [10] when large volumes of rainfall occur within a month. In our study, the highest ARP22 occurred at the onset of the rainy season, when soils were dry, which are conditions conducive to high runoff. The vertic properties of the soils probably exacerbated this. The importance of the water holding conditions of the soil on the balance between the magnitude of rainfall runoff and infiltration may also be one reason for the large spread found in the responses between cumulative rainfall (ARP22) and ANPP in this study.

The number of rain events is a metric that corresponds with the temporal distribution of rainfall and is related to the length of the dry interval, given that fewer events will always result in longer dry intervals. Although the interval between rain events could be expected to be a more direct indicator of the effect of spacing between rain events [14], in common with several other studies, we found that the number of rain events was significantly correlated with ANPP [14], [35]. ANPP responded positively to more frequent and more evenly distributed rainfall events in both vegetation types. Also the response of ANPP to the number of rainfall events is likely to be in close correspondence with the properties of the soils in the study site. As indicated above, the soils of the area have strong vertic properties which cause contraction and cracking when drying. More frequent and evenly distributed rainfall will not only maintain more even levels of water supply for the vegetation, but may also have an effect in maintaining less stressful soil physical conditions for root growth. In contrast, the size of each event was less important; as we found no relationships between either the size of the rainfall event or the interval between rainfall events and ANPP. As discussed above, this lack of response could also be related to low infiltration rates and high run-off losses when large amounts of rain fall in a short time period [36]. These findings highlight the importance of understanding rainfall distribution patterns as drivers of ecological processes in seasonally dry ecosystems.

### Evaluating Effects of Pastureland Type on ANPP and Seasonality

ANPP is a fundamental property of ecosystems because it determines the amount of energy that is made available to other trophic levels, which in pastoral systems include livestock. ANPP assessments in tropical seasonal grasslands are scarce [1]; in the grey literature [4], and where attempted, proxies such as standing biomass have often been used. The annual ANPP values measured in this study are within the ranges recorded for similar grasslands, savannas and sown pastures in the Neotropics [4], [9], [37] (Table 2). Under the pasture management applied in the study sites, we found no differences in annual ANPP between the *Brachiaria brizantha* pastures and the grasslands.

**Table 2:** Annual above-ground net primary productivity.

Grassland type and Pasture	Region, Country	Annual ANPP g m^−2 ^yr^−1^	Mean annual rainfall (mm)	References
Seasonal and humid savanna: savanna vegetation	Calabozo, Venezuela	1121	1252	San Jose and Medina 1976 after [9]
Seasonal and humid savanna: savanna vegetation	Calabozo, Venezuela	700–1100	1839	[4]
Sub-humid ecosystem: pasture*Hyparrhenia rufa*	Cañas, Costa Rica	1378	1538	Daubenmire1972, cited by [4]
Sub-humid ecosystem: pasture*Brachiaria brizantha*	Cañas, Costa Rica	1560	1500	[37]
Seasonal dry ecosystem: pasture *Brachiaria brizantha*	Muy Muy, Nicaragua	955	1547	This study
Seasonal dry ecosystem: seminatural grassland	Muy Muy, Nicaragua	898	1547	This study

The estimated annual above-ground net primary productivity for grasslands and savannas in the neotropics and for this study.

The similarity of annual ANPP amongst vegetation types is probably a result of two counteracting properties of the vegetation: the attributes of the dominant species and the complementary attributes of other species in the plant assemblage. *Brachiaria* cultivars are expected to be more productive and to have higher resource uptake and evapotranspiration than semi-natural grasslands [2], [38] commensurate with a growth form indicative of high growth rates and rapid resource acquisition strategies [39]. Erect growth has been related to high growth rates and correspondingly high rates of resource use, whilst prostrate growth form, which is characteristic of the dominant species in the grasslands, has been associated with lower productivity [40], [41]. *Brachiaria brizantha*, cvs. Marandu and Toledo are tolerant to mild water stress and the sown pastures have higher forage production than native grasses under such conditions in the Neotropics [38]. These characteristics agree with our findings. As predicted, in the rainy season, the sown pastures dominated by *Brachiaria* seem to have utilized rainfall more rapidly (steeper ANPP increase at the start of the rainy season) and also more efficiently; initially producing more biomass than grasslands per unit rainfall and reaching higher yields before ANPP starts to decline. Accordingly, the attributes of the dominant species in the vegetation appear to be an important determinant of wet season ANPP, although differences in management of sown pastures and grasslands in terms of the amount of residual biomass after grazing, may also contribute to their different responses. Although not quantified, the amount of biomass that remains after grazing events, a property of the pasture directly related to plant growth and the capacity to recover from tissue loss, is likely to have been larger in sown pastures than in grasslands, since farmers tend to manage sown pastures less intensively (SO personal observation).

Despite more rapid growth in the rainy season, sown pastures appear to stop growth more abruptly at the end of the rainy season and, during the dry season, low rainfall translated into significantly lower levels of ANPP than in grasslands. We speculate that this pattern may be attributed to the occurrence of species in the grassland assemblage with attributes that enable them to maintain growth when water supply becomes limiting. Both the sown pastures and the grasslands were very low in productivity in the latter part of the dry season which is consistent with the widespread practice of retaining trees in pastures in seasonally dry climates to provide some nutrition for livestock from tree leaves and fruits in the dry season [42].

### Temporal Stability

The result of a more defined peak of production of the sown pastures in the rainy season compared with the grasslands is also reflected in higher values of temporal stability and lower within-season coefficients of variation of ANPP for the grasslands than for the pastures. These results coincide with those reported about higher temporal stability of above-ground biomass in grasslands than in sown pastures [43], [44], and further demonstrate that grasslands stabilize function in time more than sown pastures. The higher stability in grasslands is consistent with the presence of a larger number of species and probably more functional diversity [45]. It could be expected that higher species diversity in grasslands will encompass a wider set of adaptive traits, such as diverse vegetative and reproductive phenologies and root depths, compared with sown pastures. Some further supporting evidence of the different functional stability of the two types of pastureland is their apparent difference in sensitivity to rainfall fluctuations. Although the interaction term vegetation type x number of rainfall events was not significant in the overall ANPP model, ANPP was more sensitive to the number of rainfall events in sown pastures (NRE _n = 44_ *p* = 0.0045) than in grasslands (NRE _n = 60_; *p* = 0.1914), but the large variability in the ANPP data preclude more definite conclusions.

### Implications

Understanding the outcome of predicted rainfall distribution in climate change scenarios remains a significant challenge for predicting the amount and the seasonal variability of ANPP for grasslands but more so for sown pastures in relation to their higher sensitivity to rainfall amount and its distribution. The introduction of sown pastures in the area of the study, but maintaining the traditional management does not appear to be an effective means to increase ANPP, since there were no significant gains on an annual basis. Furthermore, ANPP of sown pastures was more variable within seasons than for grasslands, and there was an indication that they were more sensitive to variation in the distribution of rainfall. Sown pastures were slightly more productive in the rainy season than grasslands, and the surplus produced in this period could be used in the dry season through harvest and storage. If this practice is not implemented, less even seasonal production is likely to impose greater challenges in grazing management for sown pastures than grasslands. Overall, the gains of replacing grasslands with sown pastures are questionable, and need to be weighed against increased costs and the ecological and environmental risk of reducing the diversity of vegetation. These suggestions are based entirely on above ground biomass but there could also be differences in nutritive value between sown pastures and grasslands. Both sown pastures and grasslands were equally unproductive in the latter part of the dry season, vindicating the widespread local practice of retaining trees in pastures.

## Materials and Methods

### Site Description

The study was conducted in the Río Grande de Matagalpa watershed in Central Nicaragua (12°31-13°20′N; 84°45-86°15′W). The area is located in Muy Muy County, in sites within an altitudinal range of 200 to 400 m. The predominant land-use is livestock farming with relatively homogeneous livestock management. The natural vegetation of the region corresponds to a transitional tropical sub-humid forest [46] with semi-deciduous vegetation, and is referred to as seasonally dry tropical forest [47]. The vegetation in the study is an assemblage of native and naturalized species including grasses, herbs and woody plants. Here we use the term semi-natural grassland to refer to a pasture covered by spontaneous vegetation that grows naturally after forest clearing, or on fallow land, and which is maintained by grazing management, including fencing and weed control.

There is a clear contrast between rainy and dry periods. Rainfall recorded between November/December and April/May is usually less than 10% of the normal annual rainfall (1971–2000) for Muy Muy (1547.1±125 mm) and the annual mean air temperature is 24.3°C [48]. Topography is undulating, with slopes between 5–45%. The bedrock consists of Tertiary volcanic tuff, a type of pyroclastic rock. Tuff in the area seems to be impermeable, which explains the limited infiltration often observed in flat areas during the rainy season. In most flat areas, it is common to find soils with vertic properties, while on steeper slopes soils are more variable. The dominant soil type in the studied sites was a greyish to black Vertisol with high organic matter content in the upper horizons (8%) a clayey subsoil, pH between 5.8 and 6.8 in the topsoil, high in Ca, Mg, and K contents, but relatively low in P, Olsen-P <10 ppm (Nieuwenhuyse *et al*. unpublished data). No specific permits were required for the described field study; however where work was carried out on private farm land (the entire study) permission to do work was granted by the owners of the land.

### Study Design

The study was planned as a randomized design for two types of vegetation cover: semi-natural grasslands, consisting of various species which share dominance, mostly prostrate grasses of the genus *Paspalum* and *Axonopus*, and sown pastures dominated by *Brachiaria brizantha* either cvs. Marandu or Toledo (cultivars of an African species used to improve pasture productivity in Tropical America). We first identified 20 areas with grasslands and pastures that were at least 10 and 3 years old, respectively, and that were managed with grazing, hand weeded at least once a year, and where no fertilizers were applied. At a second stage, five areas with grasslands and five with pastures were randomly selected from this set to establish the sampling plots. However, one pasture plot needed to be removed from the study because of a change in the use of the land. Each of the five grassland plots was fenced with an area of at least 3200 m^2^ while the four sown pasture plots were paddocks of 5000 to 6000 m^2^. All identified plots were located in areas which were similar regarding their soil physical properties and topography.

Given the management of weeding in the area and in order to homogenise the amount of biomass across the plot, at the beginning of the 2007 rainy season (early June), all the plots areas were clipped and woody species taller than 20 cm were cut at ground level. All the harvested biomass was removed from the plots.

The nine plots was divided into four quadrants, where each quadrant corresponded to a cardinal point (N, S, E, and W). At each sampling period one biomass sample (1 m x 1 m) was taken from each of the four quadrants. Sampling units were assigned using randomly generated degrees (°) and distance from the central position of the plot. This procedure was repeated at each sampling period. If a subsequent randomly generated sampling unit resulted in a sampling unit falling on part of a previous one, it was discarded and a new sampling unit was established. This procedure avoided the problem of overlapping sampling units.

Four weeks after the initial clipping, all plots were grazed by cattle. The occupation period for each plot ranged between 1 and 2 days ensuring that after grazing, the sward had a relatively uniform height across the plot, at the start of the period of biomass accumulation measurement. Once the occupation period ended, biomass samples were clipped and used to estimate the biomass at the start of the ANPP estimation period (T0). Grazing was then prevented until day 22 (T1), when the above-ground biomass was clipped again to estimate the biomass increment over the growth period (T0–T1). Sequential biomass harvests after 22-day periods of grazing exclusion were conducted approximately monthly for each plot, until June 30th, 2008. In all samplings, clippings in the grassland were conducted at 0.02 m and in the pasture at 0.12 m, to reflect average remaining pasture height after grazing under the prevailing management systems.

### Above-ground Biomass Sampling and Estimation of Above-ground Net Primary Productivity

The clipped biomass was sorted by hand into green and standing dead components directly in the field, and the total green biomass was weighed immediately with a mechanical balance (± 0.1 g; Ohaus; triple beam) to avoid mass losses as a result of water loss. Once the standing biomass was sorted, and the total green biomass was weighed, the litter on the soil surface (detached dead matter) was collected by careful hand-picking in the area of each sampling unit. The litter samples were cleaned in the laboratory to discard soil particles and remnants of live components of vegetation. The dry matter content of all above-ground biomass components was estimated on a subsample of approximately 250g fresh weight which was oven dried at 65°C, until constant weight. In addition, twice a month, a randomly selected composite samples of 100 g of litter (fresh weight) taken from each of the four samples was washed by soaking in water to eliminate possible soil contamination that could affect mass and this information was used as a reference for estimating the levels of soil particles in the litter samples cleaned in the laboratory. Plot biomass (by components) was estimated by averaging the weights of the samples from the four quadrants. ANPP was estimated as the sum of the positive differences in the three biomass components collected at the start (T0) and end (T1) of each sampling period of 22 days, applying a correction for senescence and for the transference of standing dead mass to litter [4], [49].

Annual ANPP was calculated by adding the ANPP estimated for each sampling period of 22 days during the 12-month growing cycle (July–June). Given that the ANPP measurements were taken after 22 days of re-growth and that the interval between measurements periods was close to 30 days, there was at least one ANPP measurement per month per replicate during the whole study period. However, due to unforeseen difficulties in sampling, there were only 11 biomass measurements for the computation of the annual ANPP. One site with less than 11 ANPP observations in the one-year period was dropped from the analysis. In total, eight sites were included for the analysis of annual ANPP; five for grasslands and three for sown pastures. We used the community temporal stability (S) [32] to study the relationship between types of vegetation cover and temporal variability of biomass production over the dry season (December–May) and a 12 month growing cycle (July–June).

### Rainfall Data Collection

Four rain gauges were located in four villages within the study area, and rainfall data were recorded daily. Also, data from a weather station of the Nicaraguan Institute of Land Studies (INETER) located within a 5.7 km distance from the study sites were used. For six study sites, we used the rainfall data from the rain gauges and for three sites data from the weather station (Table 3). Between July 1 2007 and June 30 2008 the daily records from the four rain gauges were accumulated for each sampling period of 22 days and for each study site, the variable resulting from this is hereafter referred to as cumulative rainfall in each sampling period of 22 days (ARP22). Also the daily rainfall data in the same period were classified into rain events. Daily rainfall records that were greater or equal to 2 mm were considered as a rainfall event. In some cases a rain event coincided with one day. However, when rainfall was measured over consecutive days, these were collectively considered as one rain event. Three metrics of rainfall distribution were calculated for each biomass sampling period: the number of rainfall events (NRE), the size of the rainfall event (SRE) and the interval between rainfall events (IRE). To prevent overestimation of the size of the rainfall event and underestimation of the number of rainfall events, and divided cases with ≥3 days of consecutive measured rainfall into two events [33].

**Table 3:** Rain gauge stations where the study plots were located.

Village name	Rain gauge station	Elevation (m)	Site (plot label)	Distance to nearest rain gauge station (km)
El Coyolar	El Coyol, Farm	300	La Laguna	0.9
			El Genízaro	2.3
			El Marandú	3.7
El Guanacaste	La Cruz, Farm	314	El Guanacaste	0.8
El Corozo	San Felipe, Farm	378	El Mango	2.1
Maizama Adentro	La Lucha, Farm	280	El Mono	1.2
Muy Muy town	Weather Station Muy Muy, 055027 (INETER)	320	Los Técnicos	2.1
			El Llano	4.5
			El Plan	5.7

Names and characteristics of villages and rain gauge stations where study plots (see plot label) were located in Muy Muy, Matagalpa, Nicaragua.

### Statistical Analysis

A multiple second-order polinomyal regression model was used to fit the relationship between ANPP and ARP22. In addition, in order to estimate ANPP-ARP22 response curves for each grassland and pasture treatment separately, a dummy variable (indicated by the treatments) and its product with the regressor variable (ARP22) variable were added to estimate a different curve for each treatment. Since the residual variance in the model increased with the mean, a power variance function depending on fitted values was added to the model. The data were collected from 9 main experimental plots, each one sampled at least 11 times. Therefore, we included ‘plot’ as a random effect to model within plot correlation (compound symmetry). Model selection was based on the Akaike Criterion.

In order to examine the effects on ANPP of season and of the two types of vegetation (grassland and pasture), we used a two-way analysis of covariance with season (rainy season 2007, early dry season 2007, late dry season 2007 and rainy season 2008) and type of vegetation as main factors, and the interaction terms. ARP22 and ARP22 squared were used as co-variables. Due to variance heteroscedasticity between seasons, a mixed model was used to carry out the analysis of variance. To perform mean comparisons we used the DGC test [50].

Annual ANPP for grassland and pasture, respectively was computed by adding the monthly ANPP estimates for the period July 1st 2007 to June 30th 2008 and the F statistics were calculated according to the model: Y_ijk_ = µ+T_i_+M_j_+TM_ij_+δ_ijk_, where, Y_ijk_ = ANPP (g m^−2^ month^−1^) of the treatment *i*, the month *j* and the replicate *k*. T_i_ and M_j_ are the two main effects (the treatment *i* and month *j* in which the measurement period occurred), and TM_ij_ and δ_ijk_, the first-order interaction and error terms, respectively.

The community temporal stability was measured as the mean biomass production of each site, divided by its standard deviation. If there were no variation, community temporal stability would be maximal (infinite). When the variation relative to the mean is large, community temporal stability is small (near 0) [51]. The community temporal stability was computed for each site through the dry season (December 2007–May 2008) and a complete year (July 2007–June 2008). In order to test for differences attributed to type of vegetation we performed an ANOVA followed by Fisher’s LSD test when necessary. All statistical analyses were done with InfoStat statistical software [50]. Estimations of general and linear mixed models were made based on lme function in the nlme package of R [52].
